# Determination of heavy metals, nitrate and nitrite in mineral and drinking bottled water in Tehran, Iran: A health risk assessment by Monte-Carlo simulation method

**DOI:** 10.1016/j.heliyon.2024.e40714

**Published:** 2024-11-28

**Authors:** Ramin Aslani, Saeideh Esmaeili, Mohamad Esmaeil Akbari, Ebrahim Molaee-Aghaee, Parisa Sadighara, Shahrokh Nazmara, Babak Mahmoudi

**Affiliations:** a Division of Food Safety and Hygiene, Department of Environmental Health, School of Public Health, Tehran University of Medical Sciences, Tehran, Iran; b Department of Food Technology Research, National Nutrition and Food Technology Research Institute, Shahid Beheshti University of Medical Sciences, Tehran, Iran; c Cancer Research Center, Shahid Beheshti University of Medical Sciences, Tehran, Iran; d Department of Food Science and Technology, School of Nutritional Sciences and Dietetics, Tehran University of Medical Sciences, Tehran, Iran; e Department of Environmental Health Engineering, School of Public Health, Tehran University of Medical Sciences, Tehran, Iran

**Keywords:** Heavy metals, Nitrate, Nitrite, Bottled water, Health risk assessment

## Abstract

Heavy metals, nitrate, and nitrite pose significant risks to public health and have raised substantial concern worldwide. This study aimed to investigate the content of nitrate, nitrite, and heavy metals, including Ba, Be, Ca, Fe, K, Li, Mg, Mn, Mo, and Na, in 30 bottled water brands in winter and summer in Tehran, Iran. Heavy metal contents in the samples were analyzed using Inductively Coupled Plasma Optical Emission Spectroscopy (ICP-OES), and nitrate and nitrite contents were analyzed using Ion Chromatography (IC). Nitrate concentrations ranged from 0.28 to 38.87 mg/L, and nitrite contents ranged from 0.001 to 0.13 mg/L. The mean concentration of Ba, Ca, K, Li, Mg, Mn, and Na in the bottled drinking water brands were 11.30, 7874.40, 121.27, 2.52, 4960.49, 0.22, and 12321.70 μg/L; and in the bottled mineral water brands were 15.71, 12262.05, 166.38, 4.13, 3747.07, <LOD, and 3156.81 μg/L. The contents of Be and Mo in all brands were below the limit of detection. Nitrate and nitrite concentrations exceeded those specified on their labels in 50 % of samples. Mg content in 4 brands and Na content in 10 brands were higher than the values listed on the labels. Furthermore, non-carcinogenic health risk assessment through bottled water consumption was estimated for Iranian children and adults. HQ values of nitrate, nitrite, Ba, Fe, Li, and Mn were less than one and acceptable. The results indicated that bottled water consumption cannot pose a significant health risk for Iranian adults and children.

## 1 Introduction

In many developing countries, water supply is affected by insufficient infrastructure and environmental pollution [[1], [2], [3]]. Unfortunately, at least 2 billion people presently reside in areas that need greater access to safe drinking water. Generally, municipal tap water and bottled water are the most commonly available options for providing high-quality and safe drinking water [[4], [5], [6]]. According to the changes in human lifestyle, such as participation in sports activities, the growing trend of eating and drinking outdoors, increased traveling, and social events, the requirement for bottled water is entirely different from tap water [7,8]. Nowadays, the popularity of bottled water is rising owing to its easy availability, low price, and transportability [6,9], so more than 350 billion liters of bottled water are consumed annually worldwide [10,11]. Furthermore, bottled water is usually balanced in minerals and free of biological contaminants, making it an ideal alternative for immune deficiency patients [7]. The most frequently used material for water packaging is polyethylene terephthalate (PET) [12], and a significant portion of 400 million PET bottles manufactured annually are assigned to water packaging [13]. These bottles can be filled with various sources, including spring water, groundwater, and municipal water [14].

Recently, concerns have been raised about bottled water quality worldwide. Bottled water safety and quality may be compromised by inappropriate packaging, transporting and storing, and leaching plastic components or additives into water [14,15]. Furthermore, most water resources contain natural and industrial-derived contaminants such as nitrate, fertilizers, pesticides, hydrogen sulfide derivatives, heavy metals, and radionuclides [16,17].

There has been evidence that the concentration of nitrates in surface waters and groundwater is increasing by human activities [18]. The daily absorption of nitrate in humans varies from 43 to 131 mg/L, and its excretion through urine is estimated between 39 and 268 mg/L per day [19]. Nitrate is relatively harmless, although nitrate in water and food may be reduced through normal flora or acidic conditions in the gastrointestinal tract into poisonous nitrite, which is called endogenous nitrite [11]. Exposure to nitrate and nitrite can contribute to methemoglobinemia (or blue baby syndrome), cancers (especially gastrointestinal cancer), thyroid dysfunction, and neurological disorders [20]. Methemoglobinemia results from converting hemoglobin into methemoglobin (through oxidizing F^2+^ present in blood cells into F^3+^) after nitrites are absorbed into the bloodstream. Infants aged 0–3 months are more susceptible to methemoglobinemia due to their normal intestinal flora. Nitrate at higher concentrations can cause this syndrome in older children and adults [21,22]. Moreover, there is the possibility of nitrite bonds with amines and amides, causing the formation of nitrosamines and nitrosamides. There are both carcinogenic and non-carcinogenic effects attributed to these compounds [4,23].

In recent decades, heavy metals in food and water have become increasingly concerning because of their toxicity, sustainability, non-biodegradation, and bioaccumulation. Some elements such as Cu, Mn, Fe, and Zn are classified as essential, and the function of specific biological processes and enzymes in the human body depends on these crucial elements. However, these can be toxic at high levels. On the other hand, some metals such as Hg, As, Cd, and Pb are inherently toxic and can potentially cause health problems [[24], [25], [26], [27], [28], [29], [30]]. Exposure to heavy metals is associated with numerous disorders, such as several types of cancer, organ dysfunctions, blood diseases and cardiovascular problems, neurological complications, and DNA damage [16,29,[31], [32], [33]]. In addition, water quality significantly depends on some elements, such as Na, Ca, Mg, and K [34]. For example, the contents of Ca and Mg are the determining factors of the total hardness of the water. Furthermore, a lack of Ca is related to osteoporosis, and Mg can diminish the frequency of abrupt death [35].

Bottled water is usually stored at higher temperatures and for prolonged durations, making monitoring and controlling it more complicated. In addition, bottled water is intended primarily for drinking but is used to produce infant formula and other food, so the presence of excessive concentrations of nitrates and nitrites in bottled water may threaten infant health [36,37]. Since bottled water sources can vary, different bottled water brands are likely to contain various concentrations of nitrate, nitrite, and heavy metals; seasonal changes also affect these levels. Therefore, it is crucial to control the chemical quality of bottled water to minimize potential health hazards caused by exposure to these contaminants, especially in summer when bottled water consumption increases outside due to warm weather. The current study aimed to (1) determine simultaneously nitrate, nitrite, and ten heavy metals, including Ba, Be, Ca, Fe, K, Li, Mg, Mn, Mo, and Na contents in 30 brands of most consumed bottled water in winter and summer, (2) compare concentrations of nitrate, nitrite, Ca, Fe, K, and Mg measured in samples with those listed on labels of bottled waters, and (3) evaluate potential non-carcinogenic health risks caused by nitrate, nitrite, Ba, Be, Fe, Li, Mn, and Mo through bottled waters consumption.

## 2 Material and methods

### 2.1 Sample collection

In total, 120 samples of 30 high-consumed bottled water brands were collected from supermarkets in Tehran, Iran. All samples were packaged in PET bottles with volumes of 0.5 L and manufactured in the winter and summer of 2022. The bottles' labels were removed to keep the anonymity of the brand names. They were coded “BDW1 to BDW15” for bottled drinking water and “BMW1 to BMW15” for bottled mineral water and were transferred to the laboratory and kept at refrigerated temperature until further analysis.

### 2.2 Nitrate and nitrite analysis

Nitrate and nitrite contents in bottled water samples were analyzed using Ion Chromatography (Metrohm 850 Professional IC) in compliance with the recommended methods by “Standard Methods: For the Examination Water and Wastewater, 22nd End” [[37], [38], [39]]. The IC column was Metrosep a Supp 4–250/4.0 for Anions, and the detector was electrical conductivity. The detection limits (LOD) were 0.14 mg/L for nitrate and 0.001 mg/L for nitrite. The detection quantifications (LOQ) were 0.42 mg/L for nitrate and 0.003 mg/L for nitrite.

### 2.3 Heavy metals analysis

Heavy metal contents in samples were analyzed utilizing Inductively Coupled Plasma Optical Emission Spectroscopy (ICP-OES) (Spectro Arcos, SPECTRO, Germany). The concentration of Ba, Be, Ca, Fe, K, Li, Mg, Mn, Mo, and Na was examined in each sample. To ensure the stability of the water components and prevent sedimentation, the samples were acidified with 1 mL of 65 % nitric acid before injection to apparatus.

Operating parameters of ICP-OES were as follows: 1400 W RF power, 14.5 L/min plasma gas flow rate, 0.9 L/min auxiliary gas flow rate, 0.85 L/min nebulizer gas flow rate, 30 rpm sample pump speed, 240 s uptake time, 60 s rinse time, and 60 s initial stabilization time. The measurement was repeated three times. A recovery study was conducted by adding different concentrations of a multi-element standard solution to the tested samples and re-measuring the sample. Recovery rates ranged from 93.817 % for Mo to 108.246 % for Na. The detection limits (LOD) were 0.098 μg/L for Ba; 0.042 μg/L for Be; 0.54 μg/L for Ca; 0.16 μg/L for Fe; 0.58 μg/L for K; 0.004 μg/L for Li; 1.308 μg/L for Mg; 0.066 μg/L for Mn; 1.666 μg/L for Mo; and 51.32 μg/L for Na. The detection quantifications (LOQ) were 0.293 μg/L for Ba; 0.126 μg/L for Be; 1.619 μg/L for Ca; 0.481 μg/L for Fe; 1.74 μg/L for K, 0.012 μg/L for Li; 3.92 μg/L for Mg; 0.199 μg/L for Mn; 4.998 μg/L for Mo; and 153.96 μg/L for Na.

### 2.4 Health risk assessment

In the current research, a Monte Carlo Simulation (MCS) was used to ascertain the health risks associated with nitrate, nitrite, Ba, Be, Fe, Li, Mn, and Mo in bottled waters sold in Tehran, Iran. Human health risk assessment calculates health risk based on exposure to contaminants through various media such as air, food, water, and soil. Since ingestion is the primary pathway of exposure to contaminants in bottled water, the non-carcinogenic health risk was assessed via ingestion for adults and children. By obtaining the chronic daily intake (CDI) and dividing it by the RFD value, the target hazard quotient (HQ) value can be calculated [15,28,32,[40], [41], [42], [43]]. Non-carcinogenic risk assessment was estimated using the following equations:

(1) $$\text{CDI}=\frac{C\times \text{IR}\times \text{EF}\times \text{ED}}{\text{BW}\times \text{AT}}$$

(2) $$\text{HQ}=\frac{\text{CDI}\;}{RfD}$$

Where C is the concentration of nitrate, nitrite, Ba, Be, Fe, Li, Mn, and Mo in bottled water, IR is the ingestion rate of water (1 L/day for children and 2 L/day for adults), EF is the exposure frequency (365 days/year), ED is the exposure duration (children = 4 and adults = 40), BW is the average body weight (15 kg for children and 70 kg for adults), and AT is the average time for children and adults (ED × EF). RfD is the oral reference dose, which for nitrate is 1.6 mg/kg/day and for nitrite is 0.1 mg/kg/day. RfD values for Ba, Be, Fe, Li, Mn, and Mo are 200, 2, 700, 28, 140, and 5 μg/kg/day, respectively [7,20,37,[44], [45], [46]].

### 2.5 Statistical analysis

The data analyses were conducted using SPSS (version 27). The data normality assessment was performed using the Kolmogorov–Smirnov test. The results were evaluated using Mann–Whitney U and Kruskal–Wallis Tests. The significance level was considered p < 0.05. The Monte Carlo simulation was performed using the Oracle Crystal Ball. The trial numbers were set at 10,000 iterations, and 95th percentile risk values were provided to highlight notable risks.

## 3 Results and discussion

### 3.1 Nitrate and nitrite concentration in bottled water

The mean concentrations of nitrate and nitrite in bottled drinking water (BDW) and bottled mineral water (BMW) in winter and summer are presented in Table 1. The tested bottled water brands contained a wide range of nitrate and nitrite concentrations. Nitrate was found in all samples except in one BMW brand in the summer (BMW6). The detection rates of nitrite in the samples decreased as follows: BDW brands in summer (93.3 %) > BMW brands in summer (60 %) > BDW samples in winter = BMW samples in winter (53.3 %). The nitrate concentration in BDW samples ranged from 0.28 to 38.87 mg/L, whereas it varied from < LOD to 10.75 mg/L in BMW samples. The maximum and minimum nitrate contents were recorded in summer in BDW2 and BMW6 brands, respectively. Nitrite concentrations in the BDW samples varied from < LOD to 0.13 mg/L, and in the BMW samples ranged from < LOD to 0.04 mg/L.

**Table 1:** Concentrations of nitrate and nitrite (mg/L) in different brands of bottled (drinking and mineral) water in winter and summer.

Brands	Bottled drinking water						Brands	Bottled mineral water				
	Summer			Winter				Summer			Winter	
	Nitrate	Nitrite		Nitrate	Nitrite			Nitrate	Nitrite		Nitrate	Nitrite
BDW1	0.89 ± 0.003	0.01 ± 0.001		6.91 ± 0.18	0.001 ± 0.001		BMW1	2.22 ± 0.002	<0.001		2.21 ± 0.01	<0.001
BDW2	38.87 ± 9.86	0.02 ± 0.002		34.03 ± 1.58	0.002 ± 0.001		BMW2	3.58 ± 0.002	0.02 ± 0.001		3.10 ± 0.89	0.002 ± 0.001
BDW3	7.52 ± 0.02	0.07 ± 0.10		8.16 ± 0.26	0.002 ± 0.001		BMW3	2.66 ± 0.004	0.02 ± 0.002		3.50 ± 0.06	0.001 ± 0.001
BDW4	3.60 ± 0.03	0.04 ± 0		4.13 ± 0.16	0.001 ± 0.001		BMW4	3.11 ± 0.01	0.02 ± 0.0003		4.77 ± 0.10	<0.001
BDW5	0.28 ± 0.004	<0.001		1.34 ± 0.02	<0.001		BMW5	10.75 ± 0.02	0.03 ± 0.001		9.96 ± 0.23	0.002 ± 0.0004
BDW6	8.42 ± 0.002	0.08 ± 0		6.41 ± 0.24	0.001 ± 0		BMW6	<0.14	<0.001		0.48 ± 0.03	0.001 ± 0.001
BDW7	5.32 ± 0.003	0.03 ± 0.001		5.10 ± 0.22	<0.001		BMW7	8.44 ± 0.02	<0.001		9.07 ± 0.23	<0.001
BDW8	7.10 ± 0.02	0.04 ± 0.001		7.76 ± 0.21	0.001 ± 0.001		BMW8	9.77 ± 0.02	0.04 ± 0.001		4.10 ± 0.12	0.001 ± 0.0003
BDW9	5.78 ± 0.02	0.04 ± 0.001		5.36 ± 0.05	<0.001		BMW9	2.23 ± 0.01	<0.001		2.08 ± 0.14	<0.001
BDW10	0.45 ± 0.01	0.04 ± 0.002		1.53 ± 0.21	<0.001		BMW10	3.15 ± 0.05	0.001 ± 0.001		3.86 ± 0.13	0.001 ± 0.001
BDW11	0.89 ± 0.004	0.03 ± 0.001		1.40 ± 0.01	<0.001		BMW11	1.33 ± 0.001	0.001 ± 0.001		2.59 ± 0.07	0.001 ± 0.001
BDW12	4.00 ± 0.01	0.02 ± 0.002		6.04 ± 0.17	0.001 ± 0.001		BMW12	1.75 ± 0.02	<0.001		2.98 ± 0.12	<0.001
BDW13	4.89 ± 0.01	0.13 ± 0.11		4.36 ± 0.07	<0.001		BMW13	3.11 ± 0.01	<0.001		4.05 ± 0.06	<0.001
BDW14	19.93 ± 0.01	0.05 ± 0.002		20.26 ± 0.33	0.002 ± 0.001		BMW14	5.32 ± 0.01	0.01 ± 0		5.20 ± 0.12	0.001 ± 0.001
BDW15	5.81 ± 0.05	0.04 ± 0.001		2.40 ± 0.18	<0.001		BMW15	2.23 ± 0.01	0.01 ± 0		2.39 ± 0.17	<0.001
INSO^a^	50	0.1		50	0.1			50	0.1		50	0.1
WHO^b^	50	3		50	3			50	3		50	3

a Iran National Standards Organization, No.6694 and No.2441.

b Codex Alimentarius, CXS 227–2001.

**Fig. 1:**
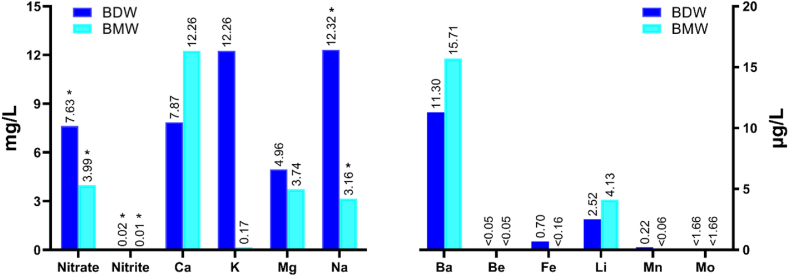
Mean concentration of heavy metals, nitrate, and nitrite in bottled drinking water (BDW) and bottled mineral water (BMW). ∗Significant difference p < 0.05.

Nitrite was generally detected at a lower concentration and in fewer brands than nitrate. The mean concentration of nitrate in BDW brands was higher than in BMW brands in both seasons. The average concentration of nitrite in BDW samples in the summer was significantly higher than in other samples. The contents of nitrate and nitrite measured in the current study were compared with INSO (Iran National Standards Organization) and WHO recommended values. The results indicated that the nitrate and nitrite concentrations were lower than the maximum allowable concentration recommended by INSO and WHO, except for the amount of nitrite in the BDW13 brand in the summer (0.13 mg/L), which exceeded the set value by INSO (0.1 mg/L). According to Fig. 1, the nitrate and nitrite contents in BDW compared with BMW were significant (p < 0.05). Also, the nitrite levels in the summer and winter were significantly different (p < 0.05). It could be attributed to human activities such as fertilizer and pesticide applications, population, and farmland usage. It is also essential to consider the temperature influences since these compounds are more soluble in water at higher temperatures, and nitrification is strongly affected. Furthermore, the increased runoff and precipitation during different months can carry more nitrate and nitrite into water sources [20,47,48].

In a study by Turhana et al. (2019), nitrate contents ranged from 0.242 to 6.123 mg/L, and nitrite amounts varied from <0.0037 to 0.015 mg/L, significantly lower than the current results [49]. Similarly, in the study by Akbari et al. (2018), the average amount of nitrate in bottled water samples ranged from 0.6 to 16 mg/L, which is lower than the present findings [50]. In agreement with our results, Cicchella et al. (2010) reported nitrate contents ranging from <0.01 to 35.1 mg/L and nitrite concentrations ranging from <0.005 to 0.132 mg/L in 186 bottled mineral water samples from Italian brands [51]. In contrast, the nitrate content reported by Al-Mudhaf et al. (2014) [52] and Dippong et al. (2019) [25], and nitrate levels in the study conducted by Miranzadeh et al. (2011) [40] were higher than in this study. In another research study conducted by Brima (2017), the mean concentration of nitrate in groundwater (11 samples), treated drinking water (13 samples), and bottled drinking water (24 samples) water was 11.82, 9.46, and 5.50 mg/L, respectively [53]. Additionally, the results of the study by Mohebali and Samari Jahromi (2013) revealed that the nitrate concentration in bottled water samples was significantly lower than its value in tap and well water samples [54]. Bertoldi et al. (2011) analyzed the chemical composition of 571 bottled mineral waters in Europe, and the results showed that 9 % of the samples contained nitrates and nitrites, exceeding European legislation [40]. Alimohammadi et al. (2018) reported that nitrate concentrations ranged from 0.146 to 50.1 mg/L, with one sample exceeding the WHO standard [37].

Nitrite in water originates from agricultural activities, such as utilizing fertilizers, organic waste, industrial releases, and waste deposits, as well as from household activities, such as leaking septic tanks and domestic water. In addition, seasonal and weather conditions can affect nitrate and nitrite levels in water resources [25]. Moreover, natural sediment erosion and sewage treatment can discharge nitrates into the water [53]. Drinking water contamination with nitrates and nitrite has become a severe public health concern worldwide. Nitrate and nitrite anions can cause methemoglobinemia and carcinogenic compounds, hypertension, cancers, congenital malformations, thyroid disorders, and goiter [55].

### 3.2 Heavy metals concentration in bottled water

The mean concentrations of heavy metals in BDW brands are demonstrated in Table 2, and those mean concentrations in BMW samples are reported in Table 3. The heavy metals detection rate was as follows: Ca, K, Mg, and Na in 30 brands (100 %) > Ba in 23 brands (76.6 %) > Li in 21 brands (70 %) > Mn in 3 brands (10 %) > Fe in 2 brands (6.6 %) > Be and Mo in 0 brands (0 %). The ascendant trend of heavy metals concentrations in BDW brands was Na > Ca > Mg > K > Ba > Li > Fe > Mn > Be = Mo, whereas in BMW brands were Ca > Mg > Na > K > Ba > Li > Be = Fe = Mn = Mo. The concentrations of Fe, Mg, Mn, and Na in the BDW brands were higher than in the BMW brands. In contrast, the amounts of Ba, Ca, Li, and K in the BMW samples were higher than in the BDW samples. Such a difference in the values of elements in bottled water is probably due to different attributes of the aquifer, such as depth of drilling, spring altitude, and characteristics of rocks the spring crosses [56]. The concentration of none of the elements exceeded the maximum allowable concentration [57]. As shown in Fig. 1, Na content in BDW and BMW brands was significant (p < 0.05).

**Table 2:** Mean concentrations of heavy metals in different brands of bottled drinking water (BDW) in summer and winter.

Brands	Heavy metals in bottled drinking waters									
	Ba (μg/L)	Be (μg/L)	Ca (mg/L)	Fe (μg/L)	K (mg/L)	Li (μg/L)	Mg (mg/L)	Mn (μg/L)	Mo (μg/L)	Na (mg/L)
BDW1	<0.1	<0.05	0.33 ± 0.01	1.4 ± 0.31	0.02 ± 0.001	<0.004	8.87 ± 0.06	0.2 ± 0.02	<1.666	3.68 ± 0.04
BDW2	0.9 ± 0.09	<0.05	4.16 ± 0.01	<0.16	0.11 ± 0.0003	<0.004	0.68 ± 0.001	<0.066	<1.666	22.18 ± 0.04
BDW3	1.2 ± 0.04	<0.05	4.09 ± 0.02	<0.16	0.11 ± 0.0001	<0.004	0.67 ± 0.0001	<0.066	<1.666	22.06 ± 0.01
BDW4	1.7 ± 0.08	<0.05	13.09 ± 0.03	<0.16	0.08 ± 0.0001	0.2 ± 0.01	4.42 ± 0.02	<0.066	<1.666	0.93 ± 0.002
BDW5	<0.1	<0.05	0.05 ± 0.01	<0.16	0.23 ± 0.0001	<0.004	7.72 ± 0.03	<0.066	<1.666	10.16 ± 0.03
BDW6	10.5 ± 0.01	<0.05	12.23 ± 0.01	<0.16	0.14 ± 0.003	1.3 ± 0.03	3.00 ± 0.006	<0.066	<1.666	2.85 ± 0.01
BDW7	10.2 ± 0.05	<0.05	9.04 ± 0.01	<0.16	0.10 ± 0.0002	0.2 ± 0.04	5.08 ± 0.006	<0.066	<1.666	10.89 ± 0.01
BDW8	58.6 ± 0.51^a^	<0.05	17.18 ± 0.08	<0.16	0.12 ± 0.001	1.8 ± 0.04	6.71 ± 0.01	<0.066	<1.666	7.93 ± 0.04
BDW9	1.8 ± 0.04	<0.05	6.78 ± 0.04	<0.16	0.15 ± 0.0001	5.7 ± 0.06	4.47 ± 0.02	<0.066	<1.666	21.18 ± 0.06
BDW10	15.3 ± 0.02	<0.05	10.46 ± 0.01	0.0 ± 0.04	0.13 ± 0.0001	2.1 ± 0.07	4.74 ± 0.01	0.1 ± 0.01	<1.666	23.32 ± 0.01
BDW11	5.7 ± 0.01	<0.05	9.17 ± 0.07	<0.16	0.15 ± 0.0004	1.5 ± 0.07	5.52 ± 0.04	<0.066	<1.666	4.69 ± 0.01
BDW12	23.05 ± 0.38	<0.05	6.03 ± 0.02	<0.16	0.23 ± 0.0002	2.8 ± 0.01	7.48 ± 0.01	<0.066	<1.666	17.92 ± 0.11
BDW13	1.38 ± 0.07	<0.05	6.01 ± 0.01	<0.16	0.14 ± 0.0003	5.2 ± 0.03	4.01 ± 0.001	<0.066	<1.666	19.34 ± 0.16
BDW14	14.55 ± 0.0	<0.05	16.42 ± 0.02	<0.16	0.09 ± 0.0002	4.4 ± 0.02	8.08 ± 0.0004	0.36 ± 0.01	<1.666	5.63 ± 0.02
BDW15	2.05 ± 0.03	<0.05	3.09 ± 0.03	<0.16	0.03 ± 0.0007	0.0 ± 0.02	2.96 ± 0.01	<0.066	<1.666	12.07 ± 0.08
WHO^a^	700	4	–	300	–	–	–	400	70	200
INSO^b^	700	–	–	300	–	–	–	400	70	200

a Codex Alimentarius, CXS 227–2001.

b Iran National Standards Organization, No.6694 and No.2441.

**Table 3:** Mean concentrations of heavy metals in different brands of bottled mineral water (BMW) in summer and winter.

Brands	Heavy metals in bottled mineral waters									
	Ba (μg/L)	Be (μg/L)	Ca (mg/L)	Fe (μg/L)	K (mg/L)	Li (μg/L)	Mg (mg/L)	Mn (μg/L)	Mo (μg/L)	Na (mg/L)
BMW1	<0.1	<0.05	3.28 ± 0.01	<0.16	0.36 ± 0.001	6.35 ± 0.001	1.24 ± 0.01	<0.066	<1.666	3.15 ± 0.012
BMW2	0.49 ± 0.04	<0.05	9.55 ± 0.04	<0.16	0.07 ± 0.0001	1.1 ± 0.005	3.64 ± 0.01	<0.066	<1.666	0.89 ± 0.01
BMW3	<0.1	<0.05	8.38 ± 0.05	<0.16	0.04 ± 0.0001	<0.004	1.86 ± 0.01	<0.066	<1.666	2.92 ± 0.01
BMW4	12.03 ± 0.12	<0.05	25.95 ± 0.13	<0.16	0.25 ± 0.0004	5.69 ± 0.04	6.77 ± 0.02	<0.066	<1.666	3.56 ± 0.003
BMW5	<0.1	<0.05	3.36 ± 0.02	<0.16	0.37 ± 0.001	6.42 ± 0.041	1.26 ± 0.01	<0.066	<1.666	3.17 ± 0.03
BMW6	10.76 ± 0.03	<0.05	23.02 ± 0.04	<0.16	0.12 ± 0.0002	1.97 ± 0.02	9.34 ± 0.13	<0.066	<1.666	3.91 ± 0.04
BMW7	11.16 ± 0.13	<0.05	18.92 ± 0.09	<0.16	0.17 ± 0.0002	4.16 ± 0.02	4.03 ± 0.01	<0.066	<1.666	12.32 ± 0.05
BMW8	6.46 ± 0.01	<0.05	14.04 ± 0.04	<0.16	0.10 ± 0.0004	<0.004	2.00 ± 0.004	<0.066	<1.666	2.68 ± 0.004
BMW9	<0.1	<0.05	3.35 ± 0.003	<0.16	0.37 ± 0.0001	6.38 ± 0.02	1.24 ± 0.01	<0.066	<1.666	3.19 ± 0.02
BMW10	55.9 ± 0.35	<0.05	9.62 ± 0.01	<0.16	0.09 ± 0.0003	<0.004	6.63 ± 0.002	<0.066	<1.666	1.22 ± 0.001
BMW11	10.9 ± 0.02	<0.05	10.90 ± 0.10	<0.16	0.05 ± 0.0003	<0.004	5.39 ± 0.01	<0.066	<1.666	0.61 ± 0.01
BMW12	13.9 ± 0.01	<0.05	11.02 ± 0.01	<0.16	0.04 ± 0.0004	<0.004	5.52 ± 0.003	<0.066	<1.666	0.55 ± 0.002
BMW13	31.1 ± 0.23	<0.05	25.64 ± 0.06	<0.16	0.09 ± 0.0001	1.7 ± 0.01	3.36 ± 0.02	<0.066	<1.666	4.99 ± 0.001
BMW14	4.4 ± 0.03	<0.05	13.55 ± 0.02	<0.16	0.06 ± 0.0001	1.2 ± 0.01	2.71 ± 0.001	<0.066	<1.666	1.06 ± 0.004
BMW15	<0.1	<0.05	3.35 ± 0.01	<0.16	0.37 ± 0.001	6.3 ± 0.01	1.25 ± 0.01	<0.066	<1.666	3.13 ± 0.002
WHO^a^	700	4	–	300	–	–	–	400	70	200
INSO^b^	700	–	–	–	–	–	–	400	–	–

a Codex Alimentarius, CXS 227–2001.

b Iran National Standards Organization, No.6694 and No.2441.

The mean concentration of Ba in samples was 11.30 μg/L in BDW samples and 15.71 μg/L in BMW samples, as well as varied from 0.1 to 58.6 μg/L in BDW brands and <0.1–55.9 μg/L in BMW brands. The highest level of Ba was found in the brands BMW10 and BDW8. The detection rate of Ba in the BDW samples was more than BMW samples. Compared to other studies, Ba contents in our study were lower than those reported by Brima (2017) [53] and Cidu et al. (2011) [58], and higher than those reported by Ungureanu et al. (2022) [56]. Ba is classified as potentially toxic at high exposure and may cause harmful effects on human health [59]. Following gastrointestinal absorption, Ba accumulates primarily in bones. Furthermore, Ba is a physiological antagonist of K, which through obstructing potassium channels, triggers extracellular hypokalemia and elevation of intracellular K. Ba poisoning causes stimulation of skeletal muscle, gastrointestinal, and cardiac, and severe exposure can lead to paralysis [60].

In all brands studied, the levels of Be and Mo were below the detection limit and were not measured. In other studies, Be and Mo levels were higher than in this study [51,53,57,61]. Be is classified as a class A carcinogen and can mimic the chemical behavior of Mg and dislocate it from specific enzymes, consequently leading to enzyme malfunction [51]. Mo is an essential element, and four enzymes in the human body require Mo, including aldehyde oxidase, sulfite oxidase, xanthine oxidase, and mitochondrial amidoxime-reducing components [26]. Mo can pose toxic effects on human health at high levels, especially in those with inadequate consumption of Cu in the diet or a Cu metabolism disorder [62].

The concentration of Fe ranged from <0.16 to 1.4 μg/L in BDW samples. The concentration of Fe in all BMW brands was lower than the detection limit. The Fe levels in other studies were higher than in the current study [25,57,63]. Fe is a crucial element for hemopoiesis and many vital functions of the body; nevertheless, high levels of exposure can give rise to health complications, such as diarrhea, nausea and vomiting, gastrointestinal ulcerations, elevated blood pressure, metabolic acidosis, and cancer [29,56,64].

Li was found in the ranges <0.004 − 5.7 μg/L in BDW brands and <0.004 − 6.42 μg/L in BMW brands. The mean concentration of Li was 2.52 μg/L in BDW samples and 4.13 μg/L in BMW samples. Brands BDW9 and BMW5 contained the highest levels of Li. Compared to our study, Li concentrations were higher in the studies executed by Dippong et al. (2020) [25], and Cidu et al. (2011) [58], and lower values of Li were recorded in the study conducted by Ungureanu et al. (2022) [56]. Long-term exposure to Li can severely damage different organs, including the kidney and parathyroid gland [56].

In BDW samples, Mn concentrations ranged from <0.066 to 0.36 μg/L. The concentration of Mn in all BMW brands was lower than the detection limit. Compared to the current study, other studies reported higher levels of Mn [25,28,57,63]. Mn is involved in the development of bones and the brain. Water containing high levels of Mn contamination can cause neurological disorders, particularly in children [65].

Ca was detected in the range 0.05–17.18 mg/L in BDW brands and 3.28–25.95 mg/L in BMW brands. The mean concentration of Ca in the BMW samples (12.262 mg/L) was higher than in BDW (7.874 mg/L). The primary source of Ca in water is limestone; however, disposal of industrial wastewater and effluents also contributes to the Ca content in water. The presence of high levels of Ca in water increases the pH up to 7.55 [25]. Mg concentrations analyzed in BDW brands varied from <0.671 to 8.87 mg/L, and in BMW brands varied from 1.24 to 9.34 mg/L. The average concentration of Mg in BMW samples (3.747 mg/L) was lower than in BDW samples (4.96 mg/L). The total hardness of water depends on concentrations of Ca and Mg. These compounds are capable of elevating the boiling point of water. Drinking water should have a hardness of 100–500 mg/L, as suggested by the WHO. Water needs to be of a suitable hardness. There is evidence that the incidence of heart disease is lower in communities that drink hard water. Soft water lacks essential minerals such as Ca and Mg, which can harm human health. Those who drink such water excrete significant amounts of Ca and Mg in urine and are more likely to develop osteoporosis, osteoarthritis, hypothyroidism, cardiovascular disorders, and hypertension. Moreover, using soft water to cook food results in losing minerals. In contrast, drinking extremely hard water containing high amounts of minerals is potentially harmful. For example, Ca in such water may deposit inside the body and result in renal or gallbladder stones [25,66,67].

The K concentrations found in the BDW brands varied from 0.02 to 0.23 mg/L and ranged from 0.04 to 0.37 mg/L in the BMW brands. The average amounts of K in the BMW samples (0.166 mg/L) were higher than in BDW (0.121 mg/L). Natural processes and water pollution are the primary sources of K in water. The solubility of K in water is lower than that of Na [25,67]. Na concentration ranged from 0.93 to 23.32 mg/L in BDW brands and 0.55 mg/L to 12.32 in BMW brands. The mean amounts of Na in the BMW samples (3.156 mg/L) were lower than in BDW (12.32 mg/L). Na is an essential nutrient that plays a prominent role in the domestic use of water and agricultural practices. However, the high amount of Na in the water can be detrimental to individuals suffering from cardiovascular disorders, renal or cardiac maladies, and hypertension [67,68]. Na and K help maintain water balance and acid-base balance in blood and tissues. Considering the limited suitable sources of K and their limited consumption, K in water provides a substantial nutritional advantage for people [66].

### 3.3 Bottled water labels

Information on labels should be clear, accurate, and consistent, and details about the product quality should be provided so the consumer can compare different brands and make an informed and confident choice. Furthermore, it is a regulatory nonconformity when the label information and claims differ from the content [69]. The label of bottled water may include different information regarding its compositions, including concentrations of Ca, Mg, Na, K, fluorides, chlorides, sulfates, nitrate, nitrite, total hardness (TH), TDS, and PH, which indicates the quality of bottled waters. The values of nitrate, nitrite, Ca, K, Mg, and Na stated on the studied bottled water labels are listed in Table 4.

**Table 4:** Nitrate, nitrite, Ca, K, Mg, and Na values (mg/L) on bottled water labels.

Brands	Bottled drinking water							Brands	Bottled mineral water					
	Nitrate	Nitrite	Ca	K	Mg	Na			Nitrate	Nitrite	Ca	K	Mg	Na
BDW1	2.4	NA	<1	0.1	22.5	1.3		BMW1	2.5	<0.005	9.6	NA	2.2	4.6
BDW2	39	<0.02	36	NA	12.5	12		BMW2	<0.8	<0.004	32.8	NA	6.4	NA
BDW3	NA^a^	NA	50	NA	12	NA		BMW3	7.1	0.014	28.3	NA	2.85	7
BDW4	3.5	0.01	38	NA	7.8	1		BMW4	2	0.01	50	1.36	6.25	17
BDW5	<1	NA	<5	1	13	16		BMW5	NA^a^	NA	12	NA	2.1	8
BDW6	0.4	0	20	0.8	10	1		BMW6	0.74	0.02	69.5	0.64	NA	7
BDW7	<2	0	35	0.4	8	9		BMW7	2.88	NA	53	NA	9	11
BDW8	NA	NA	50	NA	12	NA		BMW8	0.005	0.003	48	NA	10.20	2.76
BDW9	2.3	<0.05	11.5	NA	4.3	16.7		BMW9	NA	NA	10	NA	1.81	NA
BDW10	3.5	0.01	24.8	2	10	10.7		BMW10	0.9	0	28	NA	12.9	2.3
BDW11	1.3	0.005	32	0.7	7.68	14		BMW11	0.7	0.01	30	NA	8	6
BDW12	1	0	20	NA	15	8		BMW12	0.7	0.01	20	NA	0	6
BDW13	2.3	<0.05	11.5	NA	4.3	16.7		BMW13	0.8	0.003	81.6	1.04	5.1	4.41
BDW14	15	0	50	NA	15	9		BMW14	5	0	90	0.153	3.1	0.51
BDW15	0	0	29	0.5	1.5	32		BMW15	0.5	0	10	NA	0.3	4.5

a Not available.

As shown in Table 4, four brands concerning nitrate and seven brands regarding nitrite contents had no information on the label. The nitrate content of 65 % of samples and the nitrite content of 35 % of samples were higher than listed on labels. The amounts of Ca and K in all BDW and BMW brands were lower than those stated on the label. The concentration of Mg in brands BDW9, BDW15, BMW4, and BMW15 exceeded the values specified on the label. The greatest lack of compliance with the labels' values was observed in the case of Na, and bottled drinking water brands had the most incidents of violating their labels. Brands BDW1, BDW2, BDW6, BDW7, BDW9, BDW10, BDW12, BDW13, BMW13, and BMW14 contained higher levels of Na than their labels claimed. In healthy individuals, excess Na is excreted through the kidneys, and the appropriate balance of Na and water is regulated. However, for individuals who suffer from cardiovascular disease, hypertension, and poor kidney function, the incapacity to sustain normal Na levels may constitute a serious health risk and intake of Na should be reduced [66].

### 3.4 Health risk assessment

**Fig. 2:**
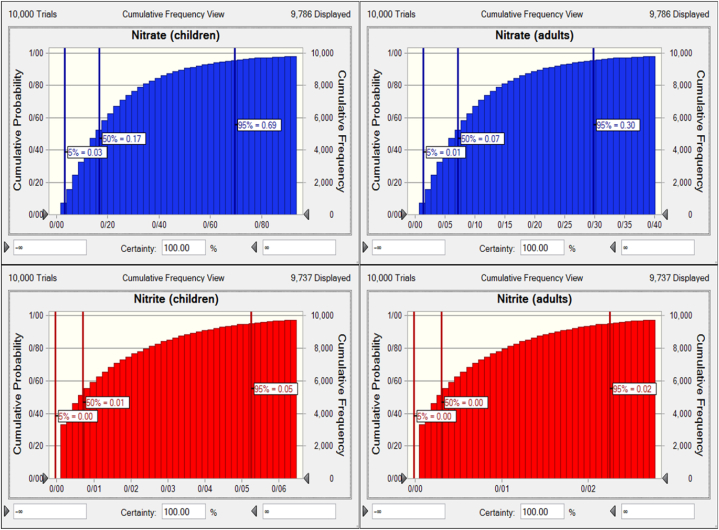
Uncertainty analysis of HQ for nitrate and nitrite through consumption of bottled water by Iranian children and adults.

**Fig. 3:**
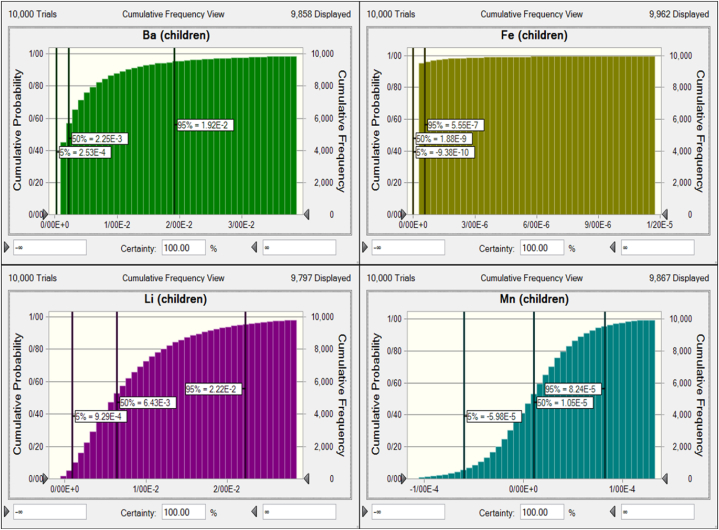
Uncertainty analysis of HQ for heavy metals through consumption of bottled water by Iranian children.

**Fig. 4:**
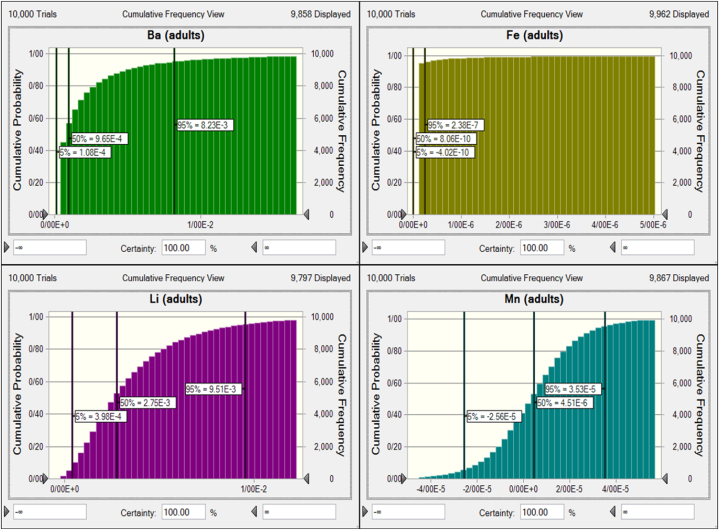
Uncertainty analysis of HQ for heavy metals through consumption of bottled water by Iranian adults.

Human health risk assessment evaluates the probability of health risks associated with different contaminant exposures from food and water consumption [15]. This study evaluated chronic daily intake (CDI) and hazard quotient (HQ) of nitrate, nitrite, Ba, Fe, Li, and Mn for Iranian children and adults via bottled water consumption. Risk assessment for Be and Mo was not calculated because the contents of these metals in all samples were below the limit of detection. The 95th percentile CDI values of nitrate, nitrite, Ba, Fe, Li, and Mn for children were 1.11, 5.26E-3, 3.84E-3, 3.89E-7, 6.22E-4, and 1.15E-5 mg/kg bw/day. The 95th percentile CDI values of nitrate, nitrite, Ba, Fe, Li, and Mn for adults were 4.76E-1, 2.25E-3, 1.65E-3, 1.67E-7, 2.66E-4, and 4.95E-6 mg/kg bw/day. HQ values of more than 1 indicate adverse health effects; if the HQ is less than 1, there is no expected potential risk to the population [3,4,28]. Fig. 2 indicates the HQ values of nitrate and nitrite for Iranian children and adults through bottled water consumption. The 95th percentile HQ values of nitrate and nitrite for children were 0.69 and 0.05, and for adults were 0.30 and 0.02. The HQ values of Ba, Fe, Li, and Mn for Iranian children and adults are shown in Fig. 3, Fig. 4, respectively. The 95th percentile HQ values of Ba, Fe, Li, and Mn for children were 1.92E-2, 5.55E-7, 2.22E-2, and 8.24E-5, respectively. Also, the 95th percentile HQ values of Ba, Fe, Li, and Mn for adults were 8.23E-3, 2.38E-7, 9.51E-3, and 3.53E-5, respectively. HQ values of nitrate, nitrite, Ba, Fe, Li, and Mn for adults and children were lower than one, and individuals would not be exposed to significant health risks, which is in agreement with previous research [57,[70], [71], [72]].

Similarly, Dippong et al. (2020) found that the HQ values of Ba, Fe, Mn, nitrite, and nitrate in bottled waters from Romania ranged from 3.0E-4 to 1.4E-1, 1.0E-4 to 1.3E-2, 2.0E-4 to 1.2E-2, 2.1E-2 to 8.2E-2, and 3.1E-3 to 9.6E-1, respectively [25]. Furthermore, Aslani et al. (2024) reported that the HQ values of Cr, Cu, Ni, V, and Zn in the mineral and drinking water distributed in different seasons in Tehran, Iran, were below one for children and adults [15]. In contrast with the present study, Ungureanu et al. (2022) reported that the mean HQ value of Fe in bottled drinking water for adults was 1.86, which was more than one and unacceptable [56]. Also, the mean HQ value of Fe for children in another study by Ungureanu et al. (2022) was 7.05 and unacceptable [73]. Olowoyo et al. (2022) found that the HQ values of Fe, Mn, and Mo ranged from 8.7E-3 to 1.3E-2, 8.6E-3 to 0.14, and 6.6E-3 to 7.8E-3, respectively [57]. Children and adults undergo different complications from pollutant exposure because of factors such as age, differences in fat tissue accumulation, differences in body weight, children's high vulnerability, and differences in daily water intake across age groups [15,20].

## 4 Conclusion

Chemical contaminants in water can threaten the health of young children, older adults, and patients with immune disorders. The current study investigated the concentrations of nitrate, nitrite, and ten heavy metals (Ba, Be, Ca, Fe, K, Li, Mg, Mn, Mo, and Na) in bottled water collected from Tehran, Iran, during winter and summer. The results revealed that all bottled water's heavy metals and nitrate concentrations were lower than the maximum allowable concentration. The nitrite content in one sample was higher than the maximum limit. HQ values for heavy metals, nitrate, and nitrite were less than one and at acceptable levels. The nitrate and nitrite content in 65 % and 35 % of the samples exceeded the bottle-labeled value. The contents of Mg in 4 brands and Na in 10 brands were higher than the values stated on the labels. Given the detrimental consequences of nitrate, nitrite, and heavy metals on health, improving drinking water treatment methods and preventive measures for water contamination, such as proper sewage management, is recommended. Moreover, the factories should be precise in listing the exact values of nitrate nitrite and other compositions on the labels. Regulatory agencies should monitor the values listed on food labels.

## CRediT authorship contribution statement

**Ramin Aslani:** Investigation, Data curation. **Saeideh Esmaeili:** Project administration, Methodology, Investigation, Conceptualization. **Mohamad Esmaeil Akbari:** Supervision, Conceptualization. **Ebrahim Molaee-Aghaee:** Writing – review & editing, Project administration, Methodology, Investigation, Conceptualization. **Parisa Sadighara:** Writing – original draft, Data curation. **Shahrokh Nazmara:** Software, Methodology. **Babak Mahmoudi:** Methodology.

## Consent to participate

Not applicable.

## Consent to publish

Not applicable.

## Data availability

All data generated or analyzed during this study are included in this published article.

## Ethical approval

IR.SBMU.CRC.REC.1400.013.

## Funding

This work was supported by the Cancer Research Center (CRC), 10.13039/501100005851Shahid Beheshti University of Medical Sciences, and project code: 27757.

## Declaration of competing interest

The authors declare that they have no known competing financial interests or personal relationships that could have appeared to influence the work reported in this paper.
